# Challenges of using tissue engineering methods in the treatment of hypospadias

**DOI:** 10.3389/fbioe.2025.1611508

**Published:** 2025-07-02

**Authors:** P. Wilczek, D. Bociaga, M. Krakos, A. Wierzbicka

**Affiliations:** ^1^ Faculty of Health Sciences, Calisia University, Kalisz, Poland; ^2^ Heart Prostheses Institute, Prof. Z. Religa Foundation of Cardiac Surgery Development, Zabrze, Poland; ^3^ Faculty of Mechanical Engineering, Institute of Materials Science and Engineering, Lodz University of Technology, Lodz, Poland; ^4^ Department of Pediatric Surgery and Urology, Hospital of J. Korczak, Lodz, Poland; ^5^ Department of Pediatric Nephrology, Polish Mother’s Memorial Hospital Research Institute, Lodz, Poland

**Keywords:** hypospodia, urethral reconstruction, biomaterials, stem cells, tissue engineering

## Abstract

**Background and objective:**

Reconstructing the urinary tract in patients with hypospadias poses a significant clinical challenge. Despite numerous surgical approaches available, outcomes remain unsatisfactory due to the complexity of the condition and the lack of standardized conventional methods. Material requirements for reconstruction are stringent, necessitating resistance to fluids, adherence, and prevention of hair growth and strictures. Materials should also be biocompatible with the ability to control their biodegradation rate.

**Methods:**

A systemic search of PubMed database for studies between 2021 and 2024 was performed. The search terms included surgical methods hypospodia, stem cells in urethra reconstruction, polymer materials used in hypospodia, preclinical and clinical study in reconstruction of urinary system, tissue engineered methods used in urinary reconstruction.

**Results:**

Treatment options are notably limited for patients requiring lengthy urethral fragments, primarily due to the scarcity of autologous tissue, particularly penile skin. There is still debate whether using a one- or two-stage surgical procedure is more appropriate. Regardless of the surgical technique used, the number of complications increases over time. It’s justify the search for new methods of urethral reconstruction Consequently, tissue engineering techniques, cell therapies, and advancements in biomaterials offer promising alternatives to traditional urinary tract reconstruction methods. However, it should be noted that at present, despite the promising results of *in vitro* studies, the translation of these studies into clinical practice is still unsatisfactory.

**Conclusion and clinical application:**

While notable advancements have occurred in tissue engineering methods, cell therapies, and modern biomaterials in recent years, the translation of laboratory findings to preclinical and clinical applications remains inadequate. This deficiency primarily stems from the absence of standardization and the relatively short duration of clinical trial follow-ups.

## Introduction

Hypospadias affects approximately 1 in 300 boys globally, with a noticeable increase in diagnosed cases within the European population over the past 2 decades. This rise can be attributed to the heightened awareness and subsequent improved detection of this male external genitalia defect (Springer et al., 2017). Delayed detection often occurs as approximately 5% of boys have a seemingly normal foreskin, revealing the abnormal urethral opening only after its retraction. Urinary tract reconstruction remains a significant clinical challenge, with approximately 300 different surgical procedures currently known, highlighting the complexity and lack of standardization in existing methods. Consequently, there is growing interest in leveraging new technologies, particularly in tissue engineering and cell therapy utilizing stem cells. Tissue engineering, an interdisciplinary field, aims to develop biological substitutes that restore, maintain, or enhance tissue function. While initial attempts at *in vitro* cell culture for urothelial cells date back to the 1980s, recent advances in tissue engineering and stem cell culture offer promising avenues for organ replacement or repair. The use of autologous cells circumvents rejection issues, ensuring regenerated tissue properties closely resemble native tissue after *in vivo* implantation. Preferred strategies in tissue engineering applications include using solely cultured autologous cells, biomaterial-based reconstructions (scaffolds or matrices), or a combination of both techniques utilizing cell-seeded scaffolds. Alternative methods seem to be very promising, but a significant problem remains the difficulty of translating laboratory test results into preclinical and clinical research results. This is largely due to the lack of standards for conducting preclinical experiments and the lack of standardized documentation of these studies. The above significantly complicates correct conclusions about the effectiveness of individual methods in the field of tissue engineering and cell therapies and, thus, the translation of the obtained results into clinical practice.

## Currently used surgical methods for the treatment of hypospadias

Currently, hundreds of surgical procedures are used to treat hypospadias (Springer et al., 2017). For distal hypospadias, a one-stage procedure is often used. The most frequently used are onlay preputial island flaps or tubularized incised plate (TIP) repair. The results of these procedures are mostly satisfactory and are not associated with a large number of complications. The situation is slightly different in the case of hypospadias located proximally. Often a two-step procedure applies in these cases. However, there is still debate whether using a one- or two-stage surgical procedure is more appropriate. The study by Lang et al. retrospectively analyzed the incidence of complications in patients with proximal hypospadias who underwent a one- or two-stage surgical procedure. It was observed that in the case of a one-stage procedure, the complication rate was 62%, and in the case of a two-stage procedure, 49%. Additionally, at least two complications were reported in patients who underwent the one-stage procedure (Long et al., 2017). The study by Pippi et al. compared various surgical techniques in the case of proximal hypospadias. The methods compared were tubularized incised plate (TIP) urethroplasty, dorsal inlay graft TIP (DIG), and staged preputial repair (SR). It was indicated that the reoperation rate for the TIP and DIP techniques was 52.6% and 52.1%, respectively, while in the case of the SR technique, this rate was 28%. The authors also point out that the use of the SR technique gave significantly better results, even though the procedure was performed in much more severe cases (Pippi Salle et al., 2016).

In treating hypospadias, not only does the surgical technique play an important role, but the tissue material used for reconstruction and its availability are equally important. Autologous transplants are often used for this purpose; autologous tissues from the genital area or from non-genital areas may be used (Altarac et al., 2012; Schwentner et al., 2011). Numerous studies indicate that if penile skin is not available, buccal mucosa may be the material of choice (Sahin and Seyhan, 2003). The use of buccal mucosa is associated with a low complication rate (Manasherova et al., 2020). It is emphasized that the buccal mucosa is easily accessible, has adequate elasticity, is mechanically resistant, and is technically easy to harvest (Dessanti et al., 2003). It should be noted that complications may occur not only in the early period after surgery but often appear later, e.g., during puberty. Regardless of the surgical technique used, the number of complications increases over time (Mousavi and Aarabi, 2014). All the above elements justify the search for new methods of urethral reconstruction, and methods using tissue engineering techniques and cell therapies seem particularly promising.

### Stem cells used in urethra reconstruction

**FIGURE 1:**
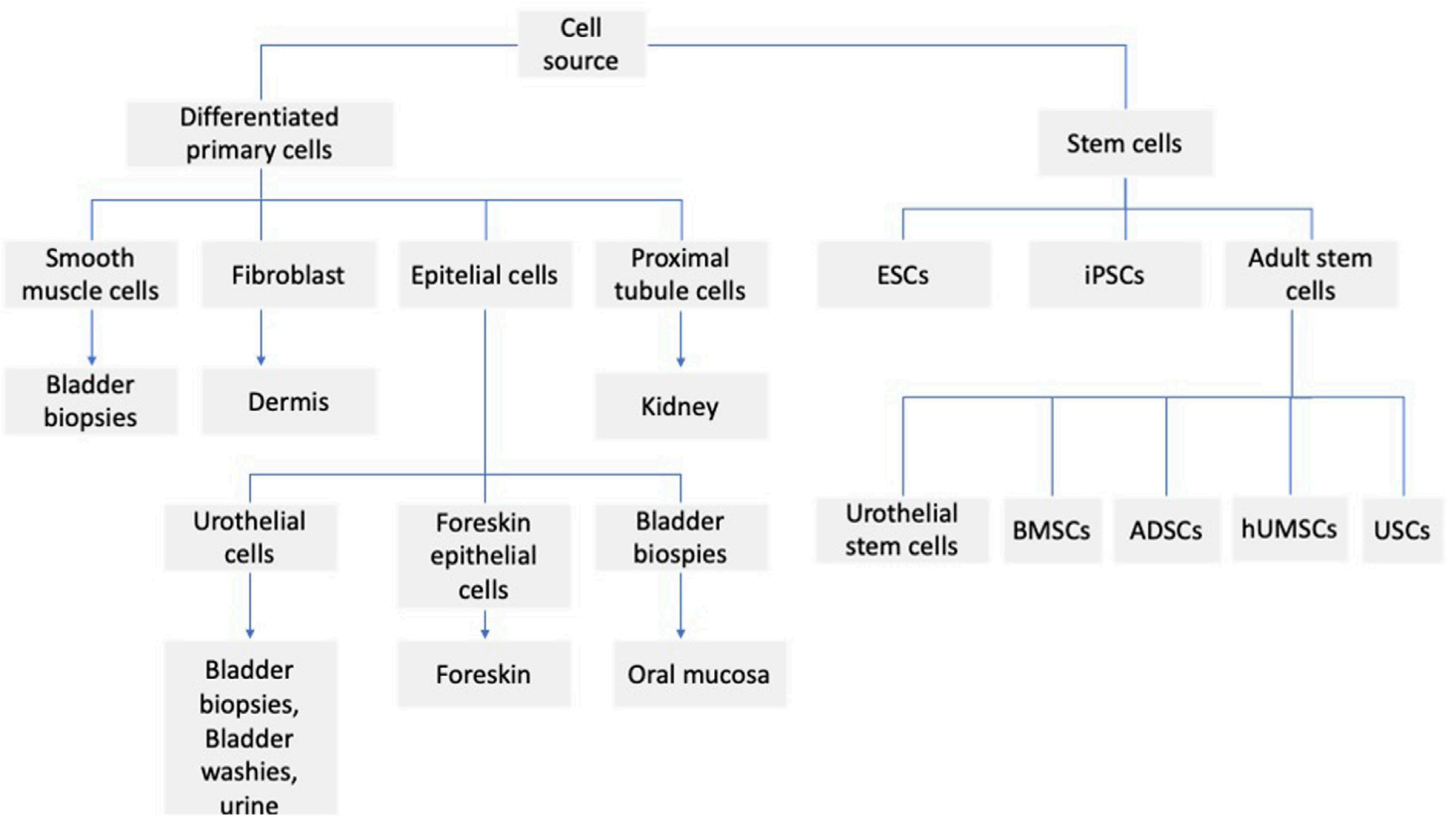
Sources of cells used in the reconstruction of the urinary system.

Various types of stem cells are used to reconstruct the urinary tract (Figure 1). Stem cells are undifferentiated cells with self-renewal potential and are capable of differentiating into mature non-regenerative cells and effector cells. According to their differentiation potential, stem cells can be divided into totipotent (zygotes), pluripotent stem cells (embryonic stem cells), multipotent stem cells (adult stem cells), and mono-potential stem cells (progenitor cells). Adult stem cells can be found in bone marrow, muscle, vascular endothelium, skin, and adipose. Therefore, more attention is paid to adult stem cells, also in applications related to urinary tract regeneration. These studies focus on both animal models and clinical trials. In the study of Castiglione et al. (2016), in the rat model, the possibility of using human adipose-derived stem cells (hADSCs) to prevent urethral stricture was assessed (US), and cells were administered topically to the site of stricture. The animals were divided into three groups: sham, US, and hADSC-treated. It was observed that the injection of stem cells prevents urethral fibrosis and elastosis and prevented functional bladder complications associated with partial outflow. It seems that this kind of strategy could also be applied to humans. It appears that TGF-b1 plays an important role in the process of fibrosis, which, in a paracrine and autocrine manner, stimulates fibrogenic cells to synthesize extracellular matrix proteins (Wynn et al., 2012). It seems that their overproduction of (Nitric Oxide) NO can be involved in the antifibrotic mechanism (Chen et al., 2005; Ferrini et al., 2010; Downey and Elborn, 2000). Such an effect is caused, among other things, by mesenchymal stem cells, which provoke paracrine inducible nitric oxide synthase (iNOS) expression and augment the antifibrotic action (Jackson et al., 2012). A similar effect is seen when hADSCs are used (Springer et al., 2017). One type of cell that can be used for urinary tract regeneration is urine-derived stem cells (UDSCs). They show a promising prospect in urethral tissue engineering. They can be easily harvested and are a promising source of autologous cells for tissue engineering techniques. In the paper of Tayhan et al. (2017), they took samples from the fresh urine of healthy patients. They characterized the isolated cells with antibodies against cytokeratin 7, CD45, and CD90, indicating the presence of MSC; some of the cells showed a positive reaction to cytokeratin 7. Epithelial cells were observed in cell culture, reaching 80%–90% confluency within 12 days. In another study by Yang et al. (2018) the cells were isolated from rabbit fresh urine. The phenotype and proliferation ability of the cells were assessed. Additionally, a differentiation experiment was performed, which was demonstrated by the ability of the cells to differentiate into smooth muscle, urothelial and osteogenic cell lines. UDSCs cells were also used in the preparation of cell-seeded scaffolds. In the study of Wu et al. (2011), a modified three-dimensional (3D) small intestinal submucosa (SIS) was cultured with human UDSCs, which differentiated into urothelial and smooth muscle cells. The cell-seeded construct was cultured under static and dynamic conditions. In the dynamic conditions, the multilayered mucosal structure was observed, and the structure was similar to the native urothelial tissue. In another study, autologous rabbit UDSCs were obtained from bladder irrigation solution samples. The cells were labeled with PKH67 to establish cell differentiation. After that, they were transplanted into rabbits to repair the ventral urethral defect. The results indicate that when UDSCs seeded SIS was used, the urethral defect could be regenerated (Liu et al., 2017). Among the disorders of the urinary system, stress urinary incontinence (SUI) is one of the most important problems. This condition mainly affects women. Currently used treatments for this problem, such as bulking agent injection and Tension-Free Vaginal Tape, have numerous limitations; therefore, great hope is associated with the possibility of using stem cell treatment (Zhou et al., 2016). The cells that are mainly used are muscle-derived stem cells (MDSCs), adipose-derived stem cells (ADSCs), bone marrow-derived mesenchymal stem cells (BMSCs), and urine-derived stem cells (UDSCs). Considering the MDSCs, they can be easily isolated as autologous cells, easily expanded *in vitro*, and then implanted into the urethral sphincter. However, the proliferation potential of MDSCs is relatively low and requires multiple injections. In the study of Lee et al. (2004), multiple injections were made into the denervated rat model, and long-term improvement of sphincter function has been observed compared to bovine collagen injection. Carr et al. reported a 1-year follow-up of women with diagnosed SUI who received MDSCs therapy. The improvement of SUI symptoms was observed in 62% of women, and in one patient, complete continence was reported (Carr et al., 2008). Despite the observed positive results, it should be pointed out that the procedure of collecting muscle tissue for cell MDSCs isolation causes great discomfort for the patient and, in the case of improper harvesting, creates a significant infection risk. Therefore, from the clinical point of view, the ADSCs seem more attractive, as adipose tissue can be easily obtained without a heavy burden on the patient. Shi et al. used ADSCs combined with silk fibroin microspheres for SUI treatment. The long-term results (12 weeks) indicate the successful restoration of function and urethral sphincter structure (Shi et al., 2014). Kuismanen et al. (2014) presented the results of the application of ADSCs in combination with collagen gel in five female patients with SUI; only two out of five patients had long-term improvement. ADSC therapy may also play an important role in the treatment of continence restoration in men following radical prostatectomy. In the study of Yamamoto et al. (2012), after ADSC injection, the bulking effect and increased blood flow were observed at the site of cell injection, which may indicate an improvement in urinary tract function, although this study involved only three patients, and certainly requires further confirmation. Another group of cells used in the regeneration of the urinary tract are the bone marrow mesenchymal stem cells. However, in this case, most studies are based on animal models, and the results are inconsistent. In the study of Corcos et al. (2011), the restoration of damaged urethral sphincter and increased improvement of leak point pressure were observed. Kinebuchi et al. (2010) injected BMDSCs in the rat model; despite the fact that the cells at the injection site survived and differentiated, as shown in histological studies, the leak point pressure outcome did not differ significantly from the control group, which was administered with cell-free medium. An important issue, apart from the selection of the right type of cells, is also the selection of the right dose of the administered cells and the supply of cells in a single or multiple injection. The paper of Janssen et al. (2019) presents the results of the research in which in the rat model in which rats underwent pudendal nerve crush and vaginal distension or a sham injury were treated with vehicle, one, two, or three doses of MSCs injection. In the case of multiple injections, cells were administered 1 h, 7, and 14 days after induction of injury. It was observed that a double or triple injection of MSCs had a significant effect on peak pressure, whereas a single injection had no such effect. Similar results were obtained by van Velthoven et al. (2010). They observed that in the ipsilateral hemisphere, after hypoxic-ischemic brain injury, the single dose of MSCs improved the sensorimotor function while the use of the second dose enhanced the effect. Lee et al. (2010) present that after partial nephrectomy, the four intravenous injections of MSCs had a beneficial effect on kidney function, and the effect was not observed after a single dose of MSCs. An interesting strategy is the co-transplantation of different cell types into the urethra. In the study of Burdzinska et al. (2018), the muscle-derived cells (MDCs) and MSCs were used. In the experiment, the aged multiparous female goats were injected with MDCs, MSCs or co-transplantation of MDC – MSC were performed. Additionally, animals treated with PBS were separated as a control group. For each animal group, the urethral pressure profile (UPP), maximal urethral closure pressure (MUCP), and functional area (FA) were measured. Immunohistochemical staining was used for the evaluation of myogenic differentiation. Improvement of parameters was observed in all groups, although the best results were obtained in the group where cell co-transplantation was used. Interesting comparative studies were also carried out using different stem cells. In the study of Kang et al. (2015), the potency of ADSCs and UDSCs was compared. In this research, both the ADSCs and UDSCs were collected from patients who required laparoscopic renal surgery. The colony formation, cell surface markers, cell proliferation, chromosome stability, immune modulation, and differentiation potential were investigated. The study indicates greater colony formation, cell surface markers, cell proliferation potential, and immune inhibition in the case of UDSCs. Also, the multilineage differentiation activity of UDSCs was higher compared to ADSCs. The UDSCs can differentiate into smooth muscle, urothelial, and endothelial cells. It also seems that the procedure for tissue material harvesting is less invasive than the ADSCs collection by liposuction. UDSCs also have the ability to secrete IL-6 and IL-8; secretion of these cytokines may inhibit T and B lymphocytes and peripheral blood mononuclear cells, which indicates the immunomodulatory effect of these cells (Zhang et al., 2014). UDSCs can also secrete pro-angiogenic factors like VEGF, which may favor the survival of the transplanted cells (Lang et al., 2013). On the other hand, there are a number of limitations associated with the use of UDSCs. Cells embedded in the scaffold lumen can also be washed out through urine and can also be damaged during a surgical procedure. In addition, cells in the first week after implantation may die as a result of the ongoing inflammatory process, ischemia, or detachment from the scaffold (Qin et al., 2014). In the case of cell therapy of the urinary tract, not only the type of cells is important, but also the supply method. Especially in the case of the reconstruction of long sections, better results are obtained with the application of a cell-seeded scaffold compared to a non-seeded scaffold (Raya-Rivera et al., 2011; Orabi et al., 2013). It is critical to use the right type of cells and to select the proper scaffold biomaterial (Ziaran et al., 2017). In the study of Bodin et al. (2010), the microporous bacterial cellulose was seeded with UDSCs, and they obtained the layer of urothelial and SMCs with cell-matrix infiltration. In the study of Wan et al. (2018), SIS was used as a scaffold, which was seeded with UDSCs. The Transwell system was used in the experiment, and the cells were cultured in dynamic conditions. As a result, the formation of multilayered uroepithelium was observed. The research also indicates cell differentiation and proper scaffold permeability. Urine is considered a profoundly cytotoxic agent, and this was confirmed in the *in vitro* studies in which urothelial and MSCs were cultured in the presence of urine (Davis et al., 2011; Adamowicz et al., 2012). This can pose a significant problem because the cells seeded on the scaffold are exposed directly to the urine. In the study of Adamowicz (Adamowicz et al., 2012), it was presented that the cells exposed to the urine survive beyond 24 h after transplantation; however, it should be taken into account that the experiment was performed at a corrected pH of 7,4. For tissue engineering applications, both decellular tissues and tissue substitutes can be used. However, it should be noted that the critical factor determining the success of tissue engineering therapy is obtaining proper cell growth on the scaffold. The first material that was used in the preclinical study was the small intestine submucosa (SIS); however, it has limited effect when used without cells (Chao et al., 2008; Dorin et al., 2008). Other material that can be used are the urethral acellular matrices. When homologous material was used, good results were obtained. Complete epithelialization and regeneration of smooth muscle cells were observed (Sievert et al., 2001). Such materials can also be used: porcine or leporine acellular bladder matrix (ABM) and acellular bladder submucosa matrix (BSM). The tissue can be seeded with autologous oral keratinocytes, ADSCs, autologous bladder smooth muscle cells, or urothelial cells (Liao et al., 2013; De Filippo et al., 2002; Chun et al., 2015). More of the research on biomaterials focuses mainly on basic or preclinical studies. However, the results of clinical trials using various types of matter are also reported. In the study of Fiala et al. (Bhargava et al., 2008), the non-cell seeded SIS was used in the reconstruction of the urinary tract. The studies included 50 patients, and the mean follow-up was 31.2 months; 80% of them had good clinical, radiological, and cosmetic results. The first study of the use of tissue-engineered oral submucosa for augmentation of urethroplasty was presented in 2008 by the Bhargava group (Patterson et al., 2011). Five patients participated in the studies, and in the initial results, rapid vascularization of the graft was observed in the entire study group. However, long-term observation indicates graft contraction and fibrosis in two patients. And complete removal of the graft in one patient (Raya-Rivera et al., 2011). In the study of Raya (Scriven et al., 2001), the PLGA (Polilactide-co glycolide) seeded with autologous bladder smooth muscle and urothelial cells was used in five boys with urothelial defect, the follow-up ranging from 36–72 months post-operatively. The improvement in urinary flow and no stricture was observed, and the graft appeared to have normal architecture 3 months after implantation. In addition to biological scaffolds, synthetic scaffolds are also used. The scaffolds differ in polymer type and form, and they can be used, for example, as membranes, films, woven, non-woven, or knitted meshes. The different forms of synthetic scaffolds may affect the seeding ability. When the fiber distance is large, the seeded cells are passed through and do not attach properly (Bisson et al., 2002). There was poor cell growth on PET (polyethylene terephthalate) scaffolds. However, when the PET was modified with the use of collagen, the improvement of urothelial cell growth was observed (Kundu et al., 2011). Comparing cell growth on composite poly-L-lactide (PLLA) film and electrospun polycaprolactone (PCL), it was observed that cell proliferation was better on the composite scaffold (Sartoneva et al., 2011). In the study of Sartoneva (Scriven et al., 1997), the synthetic scaffold composed of Poly (L-lactide-co-caprolactone) PLLCL was compared to the biological human decellularized amniotic membrane, and the biological scaffold was unsuitable for the growth of urothelial cells (UC). In contrast, it was observed in other studies that UC seeded on the de-epithelialized tissue can form a stratified epithelium (Scriven et al., 1997).

### Polymer materials used in the hypospadias surgery

**FIGURE 2:**
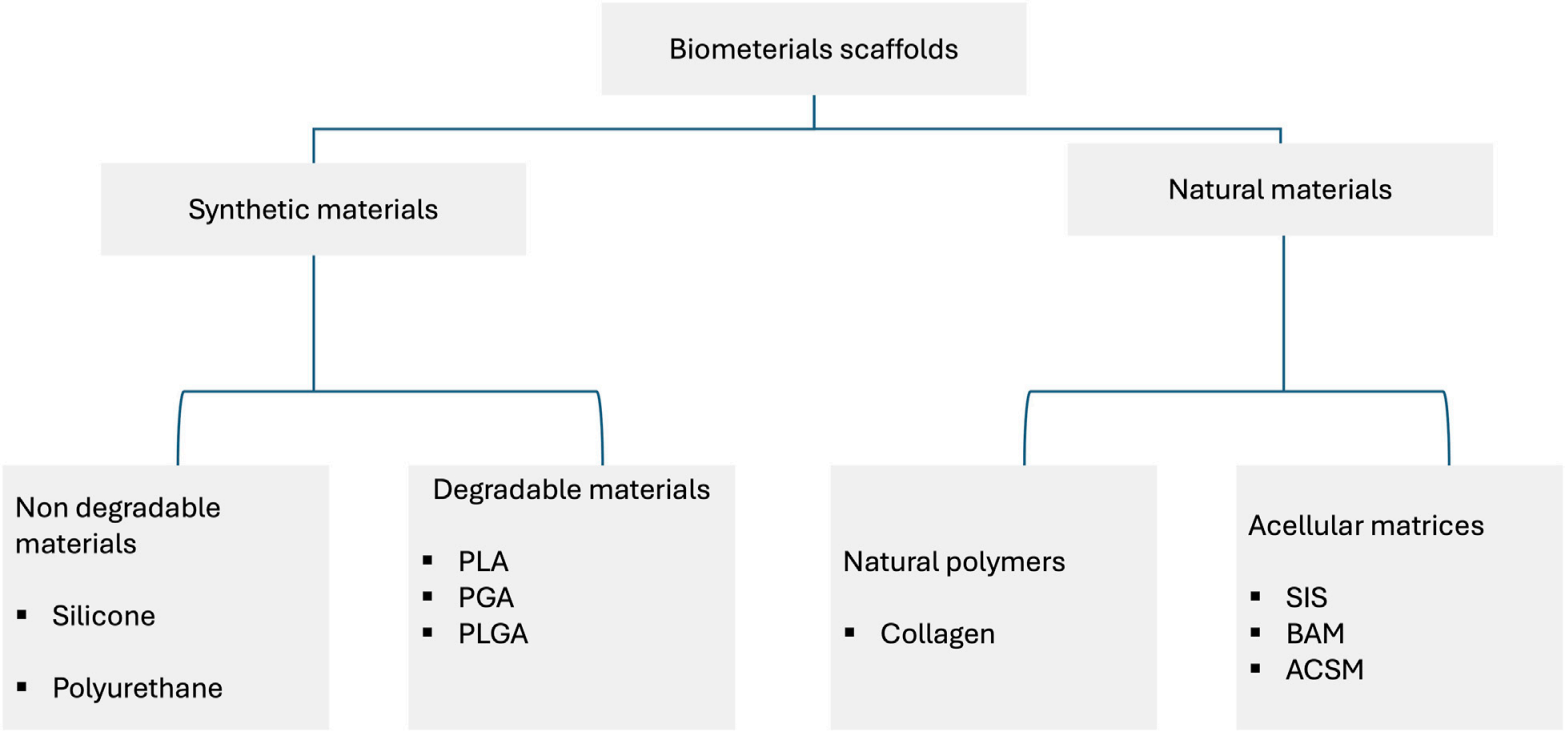
Synthetic and natural materials used in the reconstruction of the urinary system.

Currently, polymeric materials are commonly used in hypospadias surgery; they can be used as acellular stents or cell-seeded stents (Figure 2). Despite the widespread use of this type of materials, there are no detailed analyses of the properties of such materials and, consequently, the translation of these results into clinical effects. In the Rowe paper (Rowe et al., 2022), a retrospective analysis of publications from 2011–2021 was performed. The aim of the work was to compare the critical properties of materials, including thermal and mechanical analysis. Surprisingly, the study found no significant evidence that different stent materials affect the rate of surgical complications. The analysis of the publication showed that stiffer stents may reduce postoperative comfort, while a stent that is too soft and stretchy may cause problems with displacement, collapse, and fracture. Such conclusions undoubtedly encourage further search for optimal materials that could be used in the treatment of hypospadias and urethral reconstruction. Samsum’s study (Samsum et al., 2008) established an experimental model of hypospadias in rabbits. The experiment involved a transplant of autologous urethral epithelial cells cultured *in vitro*. Isolated cells were cultured on a PGA polymer scaffold, and then the polymer material with the cultured cells was implanted on the ventral surface of the penis. In the control group a polymer not seeded with epithelial cells was used. In the case of the study group, a monolayer of epithelial cells was visible after explantation, while in the control group, the epithelial cells were irregularly distributed. Atala’s publication (Atala et al., 1999) describes a clinical experiment assessing the feasibility of using a collagen-based matrix as a free graft for urethral repair. A group of four patients was selected for the study and underwent repeated correction of hypospadias using a collagen scaffold. In the long-term period after surgery, three patients achieved satisfactory clinical and cosmetic results, while the fourth patient had a complication in the form of a subcutaneous fistula. Biodegradable polymer matrices provide adequate mechanical stability in applications related to urethral reconstruction, but a significant problem is that, in many cases, they do not ensure proper cell adhesion and growth. Therefore, surface modifications of polymer materials are used to increase the adhesion potential of cells. In Bisson’s study (Bisson et al., 2002), a poly (ethylene terephthalate) scaffold was used, which was surface-modified by immobilization of collagen I and III. This modification allowed for a significant improvement in the adhesion of urothelium cells, which may certainly be important in applications related to the reconstruction of the urinary tract. Li’s manuscript (Li et al., 2014), in turn, describes an experiment in which bladder acellular matrix (BAMG) was used as a scaffold. The decelluar matrices were seeded with ADSCs cells, differentiated towards the epithelium. As a control, decellular BAMG was used. Studies have clearly shown that using cell-seeded matrices provides beneficial effects and allows for the prevention of complications such as luminal contracture and subsequent complications, including recurrent strictures. This type of research indicates that non-degradable matrices may have good mechanical properties, but due to the lack of cells, they may cause numerous complications such as calcification, fistula, chronic hematuria, encrustation, migration, and significant shortening. Undoubtedly, the interesting group of materials that may be used in the reconstruction of the urinary tract are biodegradable materials, which include, among others, poly-l-lactide (PLL), polycaprolactone (PCL), and poly (lactic-glycolic acid) (PLGA). Undoubtedly, the interesting group of materials that may be used in the reconstruction of the urinary system are biodegradable materials, which include, among others, poly-l-lactide (PLL), polycaprolactone (PCL), and poly (lactic-glycolic acid) (PLGA). They provide relatively easy control over parameters such as biomechanical properties, porosity, and degradation rate. They may additionally support cell growth. However, they require surface modification that improves cell-material interactions (Lin et al., 2015). In contrast, natural polymers have adhesive particles on their surface that promote cell adhesion and growth. One of such natural polymers may be collagen (Sabbagh et al., 1998). Other natural polymers that are used in tissue engineering are hyaluronic acid and its derivatives, alginate, and chitosan (Scriven et al., 2001). Due to its biomechanical properties, silk fibroin can also be used in urological applications. Additionally, it was observed that this material is characterized by high biocompatibility and relatively low immunogenicity (Sack et al., 2016). Decellular scaffolds are used in many areas of tissue engineering, including urethral reconstruction. For this purpose, tissues are used in which donor cells are removed using chemical, mechanical, or physical methods, leaving the extracellular matrix scaffold intact (Brown and Badylak, 2014). The decellularization process should ensure the preservation of extracellular matrix components such as collagen, elastin, fibronectin, and proteoglycans. This process should contribute to stimulating the growth of cells, including endothelial cells and smooth muscle cells (Yang et al., 2010). In Fiala’s study (Fiala et al., 2007), the usefulness of small intestinal submucosa (SIS) was assessed in the treatment of inflammatory, iatrogenic, post-traumatic, and idiopathic stenosis of the bulbar urethra and penis. A beneficial effect of the use of SIS was observed, comparable to the use of skin flaps and mucosal grafts. In turn, the work by Donkov et al. (2006) describes urethral surgery performed in nine men with strictures of 4–6 cm in diameter. This treatment used a four-layer SIS. The authors conclude that in this group of patients, the use of SIS gives good results, and the procedure is safe. However, these studies require confirmation in long-term observations. Palminteri’s study (Palminteri et al., 2012) provides interesting observations in this regard. Long-term results of the use of SIS were collected and retrospectively analyzed. The analysis shows that SIS grafts in long-term follow-up provide similar results to penile skin grafts but are less effective than buccal mucosa grafts. An important issue is tissue scaffolds seeded with cells. Cells certainly play an important role in repair processes and tissue remodeling. However, there are large discrepancies regarding the type of cells used, the method of culturing, and whether, from a clinical point of view, it is more effective to culture cells on scaffolds *in vitro* or *in vivo* (Dorin et al., 2008). The study of Corradini compared the regenerative potential of cells obtained from the urethra and oral mucosa. The cells showed a similar high proliferative potential, while clonal analysis showed differences in the ratio of stem and progenitor cells. Despite these differences observed *in vitro*, the authors conclude that both cell sources have potential in applications related to urethral reconstruction (Corradini et al., 2016). In addition to the properties of the cells, the method of obtaining the cells remains an important problem. From a clinical point of view, it is important to develop methods of obtaining autologous cells that are safe for the patient. Therefore, it is possible to use invasive methods such as biopsy or non-invasive methods. Among the non-invasive methods, the most frequently used is the isolation of cells from bladder washings (Fossum et al., 2003; Nagele et al., 2008) or from urine (Bhar et al., 2013; Liu et al., 2017).

### Clinical and preclinical challenges related to the use of tissue engineering methods in the reconstruction of the urinary system

**FIGURE 3:**
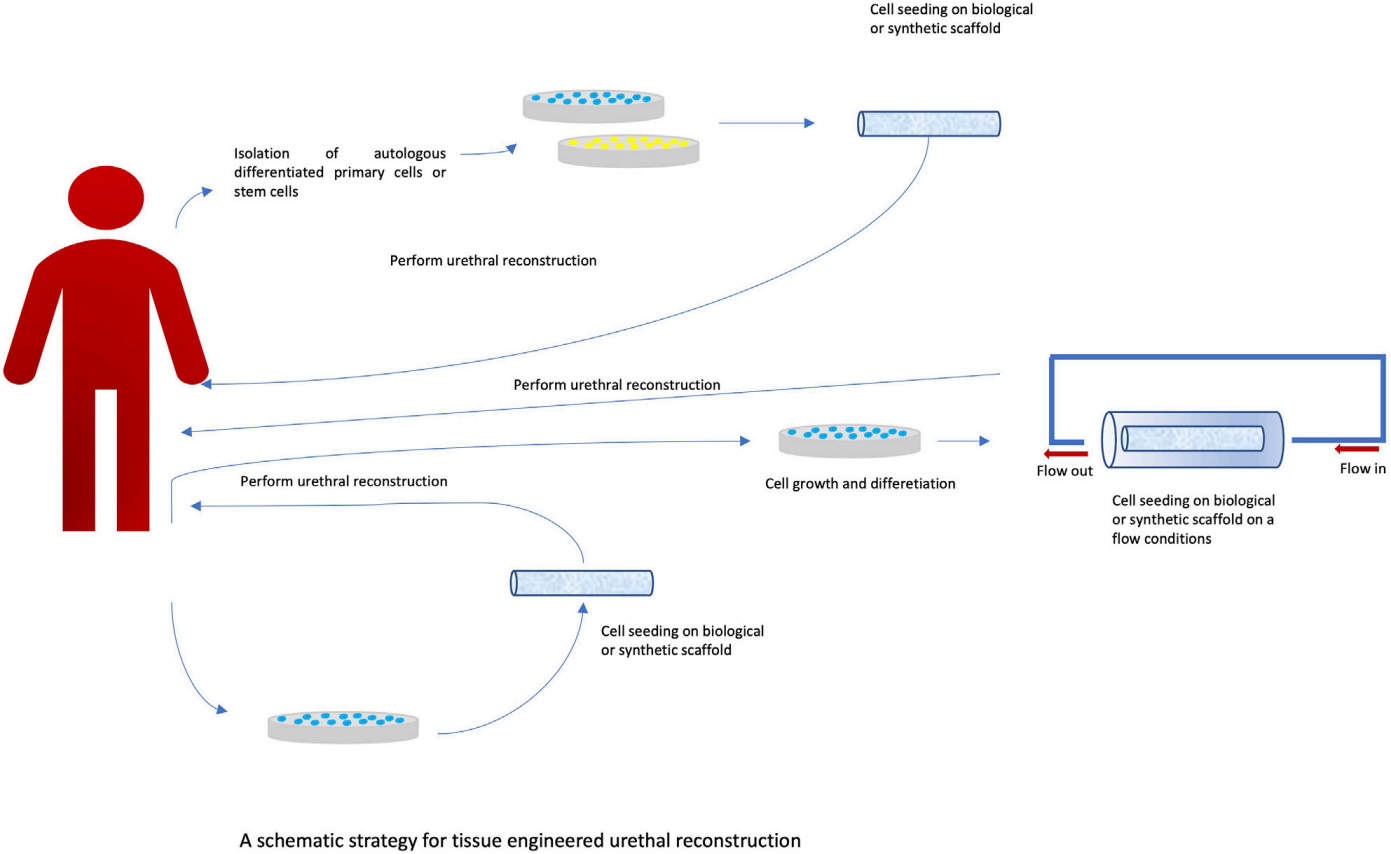
A Schematic strategy for tissue engineered urethral reconstruction.

Urethral reconstruction is still a significant clinical problem. Currently, over 300 surgical techniques are used. Such great diversity indicates the complexity of the problem and the lack of a gold standard of treatment (Figure 3). A significant problem is still the availability of appropriate tissues that could be used for reconstruction. It is possible to use skin grafts and bladder and buccal mucosa. However, all these solutions have limitations that may result in complications such as stricture formation and graft failure. Another problem is the amount of tissue that can be taken from the donor site, especially when it comes to the reconstruction of long sections. This problem is one of the reasons for the great interest in alternative methods using tissue engineering techniques. However, it should be noted that at present, despite the promising results of *in vitro* studies, the translation of these studies into clinical practice is still unsatisfactory. There are few clinical studies documenting the effects of tissue engineering treatments. These studies concern a small group of patients, and there are no long-term results. In the study by Engel et al. (2012), ten patients underwent urethroplasty. A collagen-based scaffold (MukoCell) was used, which was additionally seeded with keratinocytes. The authors conclude that the method used may be useful, but long-term observation is necessary. It is also worth noting that this type of method may help to avoid complications at the donor site. In turn, Fossum’s study (Fossum et al., 2012) assessed the long-term impact of the use of autologous epithelial cells on improving the results of hypospadias treatment. The study included six patients, and the observation period was 6–8 years. After this period, patients were assessed for their cosmetic appearance as well as functional features such as urinary function, urinary flow, artificial erection, and urethral function. The treatment method was considered safe and applicable to a selected group of patients. In another study by Bhargava et al. (2008), a buccal mucosa (TEBM) tissue scaffold was used on which autologous keratinocytes and fibroblasts were cultured to obtain full-thickness grafts. Urethroplasty was performed in a one- and two-stage procedure. Follow-up was performed at established time intervals, with the longest follow-up being 6 months. Complications such as fibrosis and spasm were noted in two of the five patients. In Raya-Rivera’s study (Raya-Rivera et al., 2011), tubular scaffolds made of polyglycolic acid:poly (lactide-co-glycolide) were used; the scaffolds were seeded with autologous epithelial cells and smooth muscle cells. Based on the conducted research, the authors indicate that this type of scaffolding can be safely used clinically and can remain functional for up to 6 years. Tissue engineering methods currently have limited clinical application. Undoubtedly, one of the obstacles associated with these methods is the relatively long time needed to prepare the transplant. Another limitation is the high cost of producing such grafts. Abbas’ manuscript (Abbas et al., 2021) reviews the literature on preclinical experiments using tissue engineering methods. The focus was on the quality of reports from preclinical experiments. The Animal Research: Reporting of *In Vivo* Experiments (ARRIVE) guidelines were used for this purpose. During the analysis of the selected publications describing preclinical experiments, numerous shortcomings were noted in the description of the methodology, including no description of the method for determining the size of the study group. The descriptions often lacked data on the conditions of the experiment and the anesthesia used. Not only do such deficiencies hinder the scientific evaluation of the publication, but they also significantly impede the translation of the results into clinical trials. One of the important problems related to conducting preclinical experiments is the selection of the appropriate animal model. The most commonly used model is the rabbit model (Versteegden et al., 2017; Qi et al., 2016). Its common use is mainly due to the similarity in the histological structure of the urethra, functional similarity, and easy surgical access (Faydacı et al., 2012; Theodorescu et al., 1998; Kemppainen et al., 1993). According to the data, the results of the experiment may be influenced by factors such as animal storage conditions (Kirk et al., 2018; Abbas et al., 2020). The type of anesthesia used is also important, especially with regard to long-term results (von der Behrens, 2014). All the above-described limitations related to conducting and reporting the results of preclinical studies make it difficult to compare the results of studies conducted in different centers. Most importantly, this makes the results of these studies difficult to translate into clinical trials, which is crucial for the proper assessment of the possibility of using tissue engineering methods in treatment.

## Conclusion

Treatment of hypospadias is a major clinical challenge. There is currently no gold standard for surgical treatment of this disease. There are currently over 300 different surgical techniques available. Unfortunately, in most cases, the use of traditional surgical techniques is associated with the risk of postoperative complications, both in the early postoperative period and in the long term. A significant problem is the lack of availability of appropriate autologous tissue that could be used in the procedure, especially in the case of surgery on long sections of the urethra. Therefore, great hopes are associated with the possibility of using alternative methods in the field of cell therapies and tissue engineering. The positive results of *in vitro* tests undoubtedly encourage this. In the treatment of hypospadias, it is possible to use various sources of autologous cells. Various materials are also available that can be used as acellular scaffolds or cellular scaffolds. Both biological and synthetic materials are used. Increasing progress is also observed in the area of bioprinting. Despite this, there are still few clinical studies on the treatment of hypospadias using bioengineering and tissue engineering techniques. One of the reasons for this is the lack of good-quality preclinical research. Preclinical research has many limitations that, in turn, make it difficult to translate the results of these studies into clinical trials. Nevertheless, the development of cell therapy methods and tissue engineering gives very promising results. Additional research and the introduction of standards for both *in vitro* tests and preclinical tests are required so that these methods can be used safely and effectively in clinical practice in the future.
